# Mass-forming renal Crohn’s disease: a case report with multimodality imaging

**DOI:** 10.1259/bjrcr.20150159

**Published:** 2016-11-02

**Authors:** Frans van Tonder, Melanie Seale, Eric Yong, Prue Hill, Jonathan Darby, Tom Sutherland

**Affiliations:** ^1^Department of Medical Imaging, St Vincent’s Hospital, Melbourne, VIC, Australia; ^2^Department of Anatomical Pathology, St Vincent’s Hospital, Melbourne, VIC, Australia; ^3^Department of Infectious Diseases, St Vincent’s Hospital, Melbourne, VIC, Australia

## Abstract

The most common extraintestinal manifestations of Crohn’s disease involve the eyes, skin, hepatobiliary tract, and the musculoskeletal and respiratory systems. Mass-forming granulomatous inflammation in extraintestinal organs is extremely rare and there are only a few reports of patients with Crohn’s disease presenting with inflammatory pseudotumours of the liver, pancreas and kidneys. We present a case of a mass-forming renal granulomatous inflammation in an adult female with Crohn’s disease. The clinical, pathological and imaging features of this case illustrate that renal inflammatory pseudotumour is a rare but important differential diagnosis of a renal mass in patients with Crohn’s disease and that radiologists should be aware of its existence when considering other more common pathologies, such as focal pyelonephritis and renal tumours. Renal inflammatory pseudotumour may have relatively non-specific imaging features and a biopsy may be required to make the diagnosis.

## Case report

The most common extraintestinal manifestations of Crohn’s disease involve the eyes, skin, and the hepatobiliary, musculoskeletal and respiratory systems.^1^ These include conditions such as uveitis, episcleritis, erythema nodosum, pyoderma gangrenosum, sclerosing cholangitis, sacroilitis and organizing pneumonia. Mass-forming granulomatous inflammation in extraintestinal organs is extremely rare, with only a few recent reports of patients with Crohn’s disease presenting with inflammatory pseudotumours in the liver, pancreas and kidneys.^2–4^ We present a case of mass-forming renal granulomatous inflammation in a patient with Crohn’s disease. A 31-year-old female was diagnosed with Crohn’s disease based on characteristic clinical features, and colonoscopy and histology of small and large bowel demonstrating non-caseating granulomas. She had undergone an ileocolic resection in 2009, following which she was treated with azathioprine and intermittent prednisolone.

**Figure 1.**
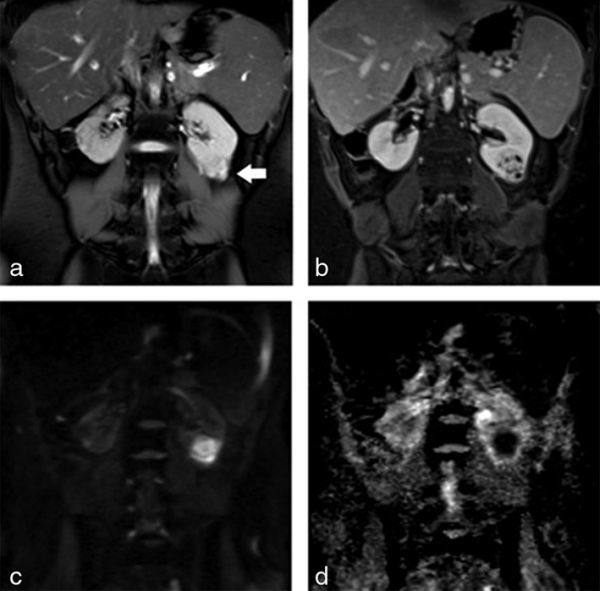
(a) Coronal half-Fourier acquisition single-shot turbo spin-echo (echo time 81 ms, repetition time 1000 ms) image of a 32-year-old female with Crohn’s disease shows the lesion is hypointense compared to normal renal parenchyma, except for a small isointense focus (arrow). (b) Coronal contrast-enhanced *T*_1_image (echo time 1.4 ms, repetition time 3.8 ms) shows enhancing septa around the non-enhancing elements, giving a honeycomb-type appearance. (c, d) The mass has high signal on b800 diffusion weighted imaging and low signal on the apparent difffusion coefficient map.

**Figure 2.**
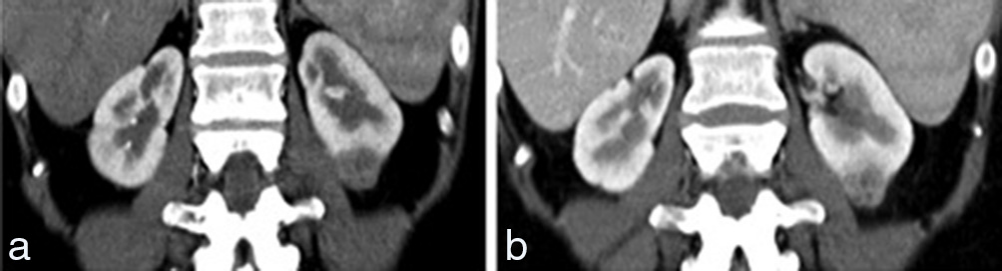
Coronal CT scan of a 31-year-old female with Crohn’s disease shows the left renal mass is homogeneously hypoenhancing in the arterial phase (a) and demonstrates patchy enhancement in the portal venous phase (b) with some hypodense internal foci.

**Figure 3.**
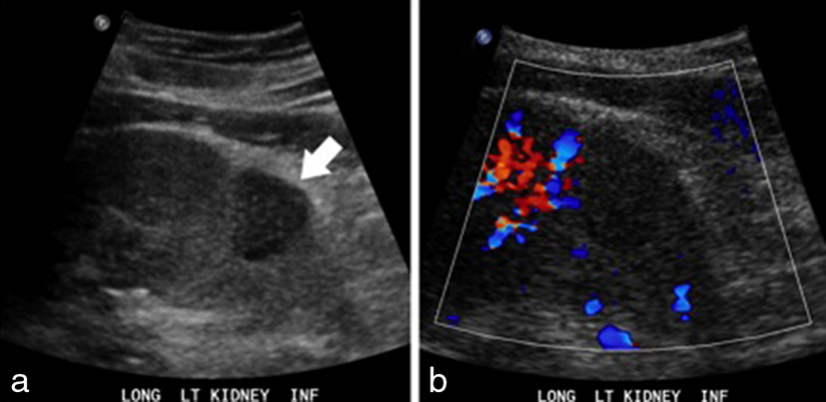
A B-mode longitudinal ultrasound image showing a homogeneous hypoechoic mass (arrow) bulging the renal contour (a) with no appreciable internal flow on Doppler (b).

MRI was performed in February 2012 for investigation of a 2-week history of abdominal pain and diarrhoea, demonstrating active enterocolitis and also revealing an incidental left renal mass that was a new finding since a CT scan of the abdomen performed in October 2011. The focal mass was located in the inferior pole of the left kidney and measured 2.5 × 2.9 × 2.3 cm; it had a lobulated margin and homogeneous signal, being slightly hypointense to the renal cortex on both *T*_2 _half-Fourier acquisition single-shot turbo spin-echo and true fast imaging with steady-state free precession images (Figure 1). The mass was isointense on pre-contrast *T*_1 _volumetric interpolated breath-hold examination and following contrast showed a honeycomb-type appearance with irregular enhancing septa around small non-enhancing elements that measured between 1 and 5 mm (Figure 1). The lesion had restricted diffusion and there was no abnormality within the perinephric fat or renal vessels (Figure 1). As the renal mass was an incidental finding on MRI focused on the small bowel, a dedicated contrast-enhanced renal CT scan was performed. On CT scan, the mass was isodense pre-contrast and homogeneously hypoenhancing in the arterial phase, while in the portal venous and delayed phases, it was hypoenhancing with cystic type non-enhancing foci within. No calcification or adenopathy was seen (Figure 2). Sonographically, the mass was slightly hypoechoic and had no demonstrable vascularity (Figure 3).

**Figure 4.**
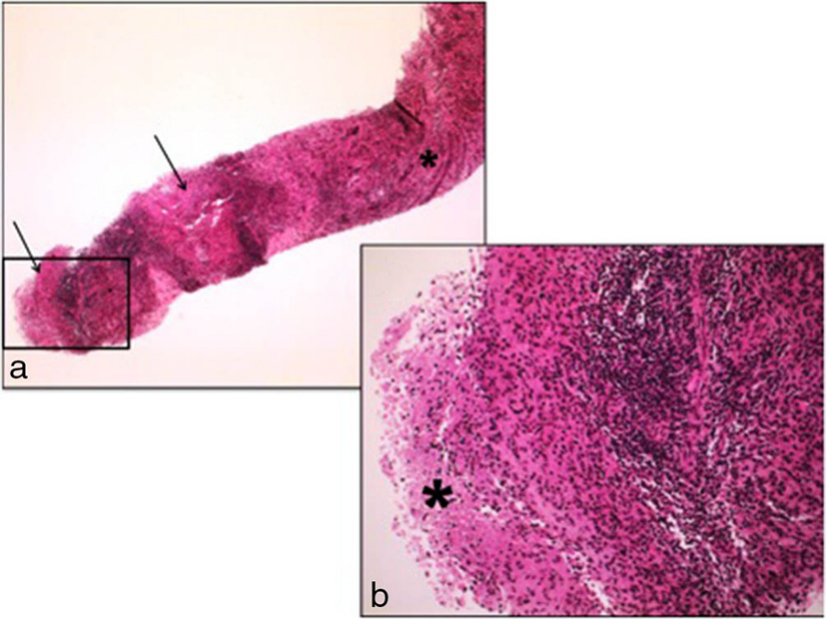
(a) The core of kidney parenchyma shows a few preserved medullary renal tubules (asterisk). The remainder of the core shows a dense mixed chronic inflammatory cell infiltrate with two foci of granulomatous inflammation with focal necrosis (arrows). Haematoxylin and eosin stain, original magnification ×40. (b) Higher magnification of the boxed part of image (a) shows some palisaded histiocytes with an area of necrosis (asterisk). No polarizable foreign material is present. Special stains for organisms (periodic acid–Schiff and Ziehl–Neelsen) were negative. Haematoxylin and eosin stain, original magnification ×200.

**Figure 5.**
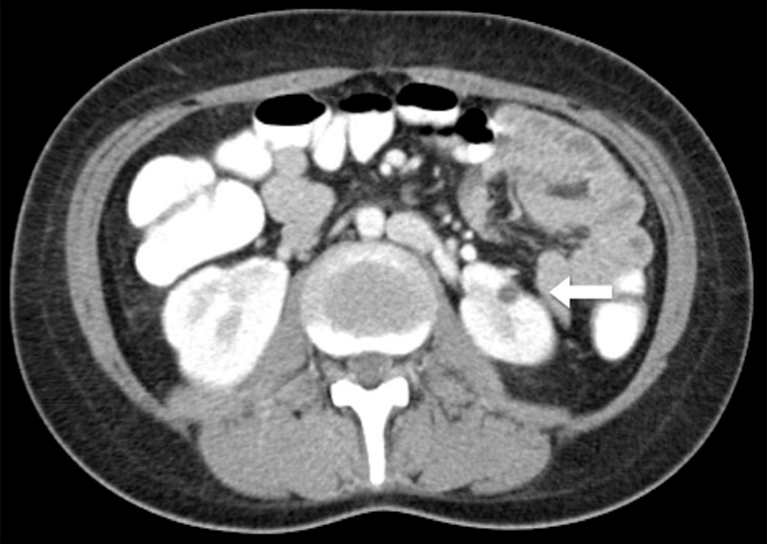
Axial CT scan in the portal venous phase shows a small focus of residual scarring in the left renal inferior pole (arrow) 6 months post initiation of prednisilone treatment.

The differential diagnosis was focal pyelonephritis, including bacterial and mycobacterial aetiologies, renal neoplasm and inflammatory pseudotumour. A QuantiFERON Gold assay (an interferon-gamma release assay used as a diagnostic test for latent tuberculosis infection) was performed and a negative result was obtained. The patient had no prior history of tuberculosis or known tuberculosis contact, and multiple early morning urine cultures were negative. She received a short course of empiric intravenous antibiotics followed by oral amoxicillin/clavulanic acid. However, these were stopped, as the patient was clinically well. The lesion was stable on follow-up ultrasound imaging in May 2012 and this prompted an ultrasound-guided biopsy, providing histology that demonstrated a dense, mixed chronic inflammatory infiltrate with foci of granulomatous inflammation with focal necrosis. Some palisaded histiocytes with areas of necrosis were seen but there were no giant cells or polarizable foreign material present. Special stains for organisms, including periodic acid–Schiff and Ziehl–Neelsen, were negative and there were no features of malignancy (Figure 4). *Mycobacterium tuberculosis* polymerase chain reaction on tissue biopsy was negative. A tentative diagnosis of an inflammatory pseudotumour was made and a course of 30 mg daily of prednisolone was commenced, resulting in resolution of the lesion, with only a small focus of scar present on follow-up ultrasound and CT imaging performed in October 2012 (Figure 5). This response confirmed the diagnosis of inflammatory pseudotumour associated with Crohn’s disease.

## Discussion

### Aetiology and demographics

Renal involvement in Crohn’s disease manifests most commonly as nephrolithiasis, immunoglobulin A nephropathy, interstitial nephritis, arterionephrosclerosis, acute tubular injury, proliferative glomerulonephritis, amyloidosis and minimal change disease.^5–9^ Renal granulomatous inflammation, characteristic of that associated with Crohn’s disease, has been described; however, to our knowledge, there is only one other case report of a discrete renal inflammatory pseudotumour attributed to Crohn’s disease.^4^ In this case, histology of the lesion following left nephrectomy was that of nodular granulomatous inflammation, including multinucleated giant cells, eosinophils, epitheloid cells, plasma cells and lymphocyes, typical of the phenotype seen in Crohn’s disease.^4^

### Clinical and imaging findings

In our case of renal pseudotumour due to Crohn’s disease, we find a more defined mass lesion compared with a diffuse multinodular presentation in the case of literature. In contrast to the prior case report of a purely solid lesion, we see non-enhancing cystic components in the portal venous and delayed phases. MRI and ultrasound imaging obtained in our case show that the lesion is a *T*_2_ hypointense and *T*_1_ isointense mass with a honeycomb-type appearance with irregular enhancing septa around small non-enhancing cystic elements. It is a slightly hypoechoic mass with loss of corticomedullary differentiation and no appreciable vascularity.

### Treatment and prognosis

Following biopsy of the lesion, the differential diagnosis was narrowed down to Crohn’s inflammatory pseudotumour and tuberculous pyelonephritis, which was subsequently excluded based on negative tuberculosis culture and polymerase chain reaction, negative immunohistochemical staining and a radiological response to immunosuppressive treatment.

### Differential diagnosis

The principal differential diagnosis of a renal parenchymal mass in a patient with Crohn’s disease is focal pyelonephritis, renal tumour and inflammatory pseudotumour. Focal pyelonephritis can be caused by bacterial, fungal and mycobacterial organisms, and in addition to the clinical presentation, imaging features such as enlargement of the kidney, perinephric stranding and collection or abscess formation may avert the need for biopsy.^10^ Perinephric space spread, renal vein invasion, and local or distant metastases are imaging features that may differentiate renal tumour and histology thereof is diagnostic.^11^ Wegener’s granulomatosis can be associated with Crohn’s disease and can rarely present with a renal inflammatory pseudotumour; however, it can be differentiated from direct renal manifestation of Crohn’s disease by necrotizing granulomas and vasculitis seen histologically.^12^

## Learning points

1. The clinical, pathological and imaging features of our case confirm that inflammatory pseudotumour is a rare but important differential diagnosis of a renal mass in patients with Crohn’s disease.
2. Biopsy may be required owing to the relatively non-specific imaging features and samples should be sent for culture, as focal pyelonephritis is a far more common differential that requires exclusion.

## Consent

Written informed consent for the case to be published was obtained from the patient for publication of this case report, including accompanying images.
